# Global Transcriptomic Analysis of the Interactions between Phage φAbp1 and Extensively Drug-Resistant Acinetobacter baumannii

**DOI:** 10.1128/mSystems.00068-19

**Published:** 2019-04-16

**Authors:** Zichen Yang, Supeng Yin, Gang Li, Jing Wang, Guangtao Huang, Bei Jiang, Bo You, Yali Gong, Cheng Zhang, Xiaoqiang Luo, Yizhi Peng, Xia Zhao

**Affiliations:** a State Key Laboratory of Trauma, Burns and Combined Injury, Institute of Burn Research, Southwest Hospital, Third Military Medical University (Army Medical University), Chongqing, China; b Bioinformatics Center, Department of Microbiology, Third Military Medical University (Army Medical University), Chongqing, China; University of California, San Francisco

**Keywords:** φAbp1, Acinetobacter baumannii, RNA-seq, bacteriophage, transcriptome

## Abstract

Acinetobacter baumannii is a growing threat, although lytic bacteriophages have been shown to effectively kill A. baumannii. However, the interaction between the host and the phage has not been fully studied. We demonstrate the global profile of transcriptional changes in extensively drug-resistant A. baumannii AB1 and the interaction with phage φAbp1 through RNA sequencing (RNA-seq) and bioinformatic analysis. Only 15.6% (600/3,838) of the genes of the infected host were determined to be differentially expressed genes (DEGs), indicating that only a small part of the bacterial resources was needed for φAbp1 propagation. Contrary to previous similar studies, more upregulated rather than downregulated DEGs were detected. Specifically, φAbp1 infection caused the most extensive impact on host gene expression at 10 min, which was related to the intracellular accumulation phase of virus multiplication. Based on the gene coexpression network, a middle gene (*gp34*, encoding phage-associated RNA polymerase) showed a negative interaction with numerous host ribosome protein genes. In addition, the gene expression of bacterial virulence/resistance factors was proven to change significantly. This work provides new insights into the interactions of φAbp1 and its host, which contributes to the further understanding of phage therapy, and provides another reference for antibacterial agents.

**IMPORTANCE** Previous research has reported the transcriptomic phage-host interactions in Escherichia coli and Pseudomonas aeruginosa, leading to the detailed discovery of transcriptomic regulations and predictions of specific gene functions. However, a direct relationship between A. baumannii and its phage has not been previously reported, although A. baumannii is becoming a rigorous drug-resistant threat. We analyzed transcriptomic changes after φAbp1 infected its host, extensively drug-resistant (XDR) A. baumannii AB1, and found defense-like responses of the host, step-by-step control by the invader, elaborate interactions between host and phage, and elevated drug resistance gene expressions of AB1 after phage infection. These ﬁndings suggest the detailed interactions of A. baumannii and its phage, which may provide both encouraging suggestions for drug design and advice for the clinical use of vital phage particles.

## Abstract (precis)

Previous research has reported the transcriptomic phage-host interactions in Escherichia coli and Pseudomonas aeruginosa, leading to the detailed discovery of transcriptomic regulations and predictions of specific gene functions. However, a direct relationship between A. baumannii and its phage has not been previously reported, although A. baumannii is becoming a rigorous drug-resistant threat. We analyzed transcriptomic changes after φAbp1 infected its host, extensively drug-resistant (XDR) A. baumannii AB1, and found defense-like responses of the host, step-by-step control by the invader, elaborate interactions between host and phage, and elevated drug resistance gene expressions of AB1 after phage infection. These ﬁndings suggest the detailed interactions of A. baumannii and its phage, which may provide both encouraging suggestions for drug design and advice for the clinical use of vital phage particles.

## INTRODUCTION

Acinetobacter baumannii is responsible for numerous health care-associated infections and burn and wound infections (1, 2). As a Gram-negative opportunistic pathogen, A. baumannii was recently listed as one of the six most dangerous pathogens due to its multiple resistance to antibiotics (2, 3). In addition, more A. baumannii strains were found to be resistant to all known antibiotics, which has alerted people to find an alternative arsenal (4, 5).

In the middle of the 1910s, the bacteriophage (phage) was suggested to have a positive outcome in the treatment of human infections (6, 7). During the following decades, increasing evidence has shown the feasibility of phage therapy to treat drug-resistant bacterial infections (8, 9). Indeed, not only has active bacteriophage been applied directly in the clinic under prudent observation (10), but also new phage-derived potential antimicrobial agents have been identified and certified (11, 12). However, phage-host interactions have not been fully studied.

Recent interest in bacteriophages has been triggered by increasing antimicrobial resistance, omics development of phage-host studies, and the screening of new antibacterial agents from phage-derived gene products (13, 14). Like all viruses, the phage relies heavily on host metabolism and must take over host processes to complete a productive infection (15, 16). Therefore, the course of phage infection is a complex struggle between the virus and the bacterial host (17, 18). However, current knowledge of phage-host interactions is based largely on a small number of Escherichia coli phages (19–21), Pseudomonas aeruginosa phages (22), and phages with less clinical importance (23), whereas insight into the phage infection courses in multidrug-resistant strains remains quite limited. Thus, an understanding of phage interactions with A. baumannii is essential for the development and application of phage therapy.

In previous reports, we screened a lytic A. baumannii phage, named φAbp1, from hospital sewage against a clinically isolated extensively drug-resistant (XDR) A. baumannii strain, AB1 (XDR-AB1), in our burn ward (12). φAbp1 was proven to be a qualified candidate for the treatment of both systemic and local XDR A. baumannii infections in mouse models (9). Moreover, φAbp1 gene product 50 (*gp50*), endolysin, was reported to exhibit marked lytic activities against 48 clinically isolated XDR A. baumannii strains with different multilocus sequence typing (MLST) types (8), implying that *gp50* can be an excellent anti-infection agent against XDR A. baumannii. However, the interactions between φAbp1 and its host are unclear, which limits its clinical application in the future. Therefore, we need an in-depth understanding of the interactions between φAbp1 and its host.

In this work, RNA sequencing (RNA-seq) was performed to investigate the interactions between φAbp1 and its host at three time points representing each infection stage. Analyses of gene expression patterns, differentially expressed genes (DEGs), and coexpression during infection were performed. This work aimed to draw new insight into the interactions between φAbp1 and its host, to establish a general understanding of φAbp1-based anti-A. baumannii phage therapy, and to provide more options for antibacterial agents.

## RESULTS

### Experimental design of RNA-seq after φAbp1 infection.

First, we investigated the resistance of the host strain AB1 to 20 antibiotics commonly used clinically (see Table S1 in the supplemental material). The results showed susceptibility to polymyxin B only, affirming the grim antibiotic resistance situation and tremendous clinical importance of φAbp1.

10.1128/mSystems.00068-19.1 TABLE S1 Antibiotic susceptibility testing of A. baumannii AB1. Download Table S1, DOCX file, 0.02 MB. Copyright © 2019 Yang et al. 2019 Yang et al. This content is distributed under the terms of the Creative Commons Attribution 4.0 International license.

**FIG 1:**
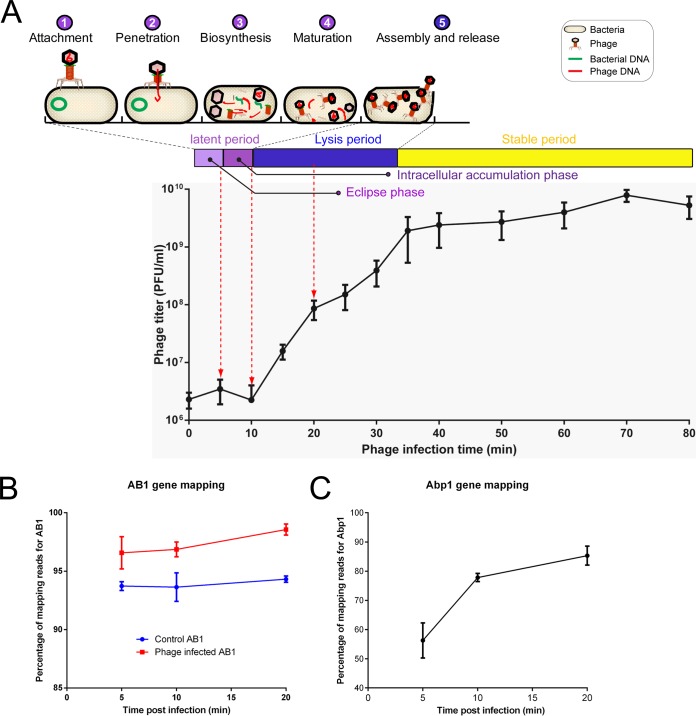
Life cycle annotation, one-step growth curve, and mapping of φAbp1. (A) The phage multiplication stages in a life cycle were divided into attachment, adsorption, penetration, biosynthesis, maturation, and assembly and release. The one-step growth curve of φAbp1 is from 0 to 80 min, which includes 3 periods: a latent period (divided into an eclipse phase and an intracellular accumulation phase), a lysis period, and a stable period. Red dotted arrows indicate the time points of sample collection. (B and C) Percentages of RNA-seq reads mapping to the reference AB1 (B) and φAbp1 (C) genomes at different infection time points.

Three sampling time points were selected for subsequent RNA sequencing according to the life cycle of φAbp1. AB1 cultures infected with φAbp1 at 5 min (eclipse phase), 10 min (intracellular accumulation phase), and 20 min (lysis period), as well as three AB1 cultures free of phage at the corresponding time points as control groups, were collected for RNA-seq analysis (Fig. 1A). RNA-seq was repeated three times for each group.

### φAbp1 takes over the transcriptional resources of host cells.

A. baumannii strain ATCC 17978 (GenBank accession no. NZ_CP018664.1) and *Acinetobacter* phage φAbp1 (GenBank accession no. NC_021316.1) were applied as the reference genomes. RNA-seq analysis generated an average of 11.8 million or 12.3 million reads in bacterial cultures infected with or without phage, respectively. The RNA-seq reads were aligned to both the AB1 and φAbp1 genomes in a strand-specific manner. The proportion of reads mapping to the AB1 genome stayed above 92%, indicating that ATCC 17978 is a proper reference for AB1 annotation (Fig. 1B). For phage consideration, the proportion of reads mapping to the phage genome increased from 56.3% (5 min) to 85.3% (20 min) (Fig. 1C), suggesting a process during which φAbp1 took over the transcriptional resources of the host cells.

**FIG 2:**
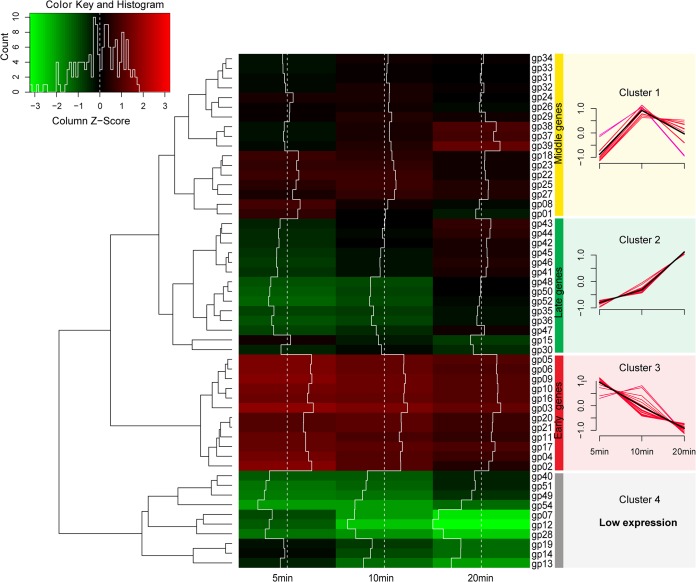
Transcriptomic profile of φAbp1 genes in the host cells. (Left) Hierarchical cluster heat map of φAbp1 genes; (right) expression level of each cluster of φAbp1 genes. Based on the FPKM values of genes, hierarchical cluster analysis was performed using ward.D2 and Minkowski methods. A total of 54 φAbp1 genes (*gp01* to *gp54*) were clustered into 4 clusters (clusters 1 to 4).

Next, 54 phage genes were clustered by hierarchical cluster analysis based on their transcriptional levels (fragments per kilobase of transcript sequence per million base pairs sequenced [FPKM] values) into 4 clusters of genes, including middle genes (cluster 1, at 10 min) (Fig. 2), late genes (cluster 2, at 20 min), early genes (cluster 3, at 5 min), and low-expression genes (cluster 4).

### φAbp1 infection induces more activation than inhibition of AB1 genes.

**FIG 3:**
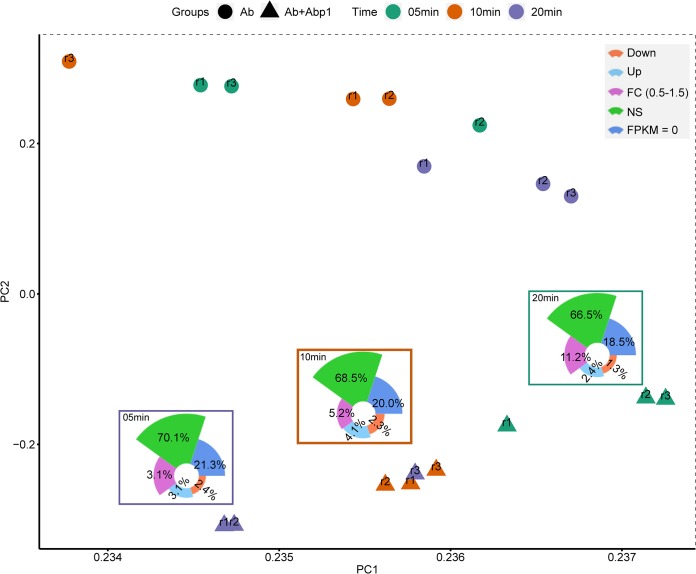
PCA plot of the expression of AB1 genes. The principal-component analysis was performed according to the expression levels of AB1 genes in phage-infected groups (Ab+Abp1) and phage-free groups (Ab). A greater distance between points suggests a greater difference in AB1 gene expression in those 6 samples. Down, downregulated DEGs; Up, upregulated DEGs; NS, nonsignificantly changed genes.

On the basis of the FPKM values of all 3,838 AB1 genes, PCA (principal-component analysis) showed that the greater distance between points suggested a greater difference in AB1 gene expression (Fig. 3), suggesting that the gene expression of the phage infection group changed far more than that of the phage-free group. According to the expression levels, genes with fold change (FC) values of >1.5 and *q* values of <0.05 were defined as DEGs. Totals of 3.7% (145/3,838), 6.4% (244/3,838), and 5.5% (211/3,838) DEGs were found 5 min, 10 min, and 20 min after φAbp1 infection, respectively. Contrary to previous similar studies (22), more upregulated DEGs than downregulated DEGs were detected in our study. Approximately 68% of the AB1 genes were expressed stably during φAbp1 infection (nonsignificant [NS] [*q *>* *0.05]), implying that only a small part of the bacterial resources was needed for φAbp1 propagation. The highest upregulation rate of the host genes was at 10 min (156; 4.1%).

### Functional analysis reveals the step-by-step control of φAbp1 on host genes.

**FIG 4:**
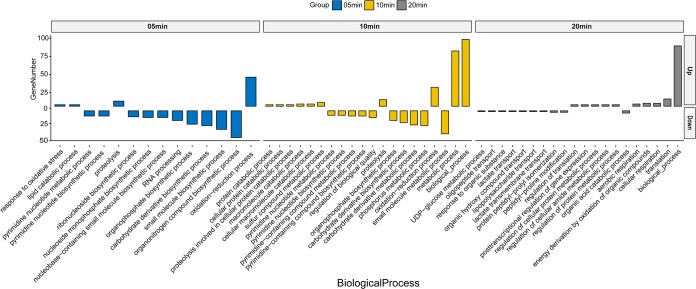
GO analysis for the biological processes of host DEGs (up- and downregulated genes). GO terms enriched from upregulated (Up) and downregulated (Down) genes are shown.

The host DEGs were grouped into the eclipse phase (5 min), intracellular accumulation phase (10 min), and lysis process (20 min) according to phage infection time points and underwent gene ontology (GO) analysis. The biological function enrichment of GO results showed that DEGs had a remarkable function classification among the three stages. The upregulated genes mainly involved stress reactions (such as oxidation-reduction processes and proteolysis) in the eclipse and intracellular accumulation phases (5 and 10 min), metabolic processes (10 min), and translation processes (20 min) (Fig. 4). The downregulated DEGs mainly included host biosynthetic processes (5 and 10 min), nucleic acid metabolic processes (5 and 10 min), and material transport processes (20 min) (Fig. 4). The detailed expression data on specific genes (>1.5-fold; *q* values of <0.05) are presented in Table S2.

10.1128/mSystems.00068-19.2 TABLE S2 Detailed GO expression data on specific genes of A. baumannii AB1. Download Table S2, DOCX file, 0.04 MB. Copyright © 2019 Yang et al. 2019 Yang et al. This content is distributed under the terms of the Creative Commons Attribution 4.0 International license.

**FIG 5:**
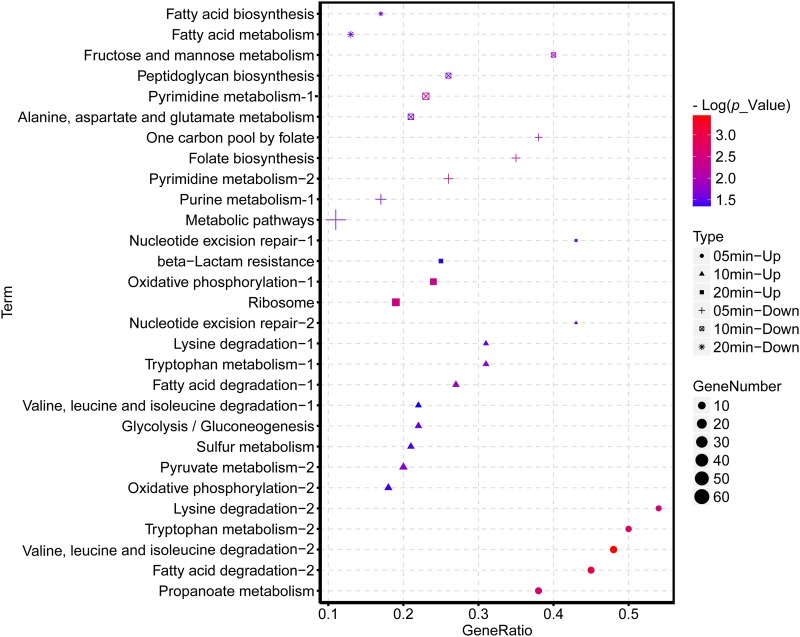
KEGG categories of host DEGs (up- and downregulated genes) enriched at selected points after φAbp1 infection. The shape of the point indicates the time points. The enrichment *P* value of each pathway was normalized as negative log(*P* value) and is shown as a color gradient. The number of genes enriched in each pathway is represented by the size of the points.

KEGG (Kyoto Encyclopedia of Genes and Genomes) pathway enrichment suggested that the downregulated genes of AB1 were significantly enriched in the pathways related to nucleic acid complements, such as purine metabolism and pyrimidine metabolism (Fig. 5). The upregulated genes were enriched in a wide range of KEGG pathways, among which the most remarkably enriched pathways were the multiple amino acid pathways at 10 min and the ribosome pathway at 20 min.

### Phage-host interaction network analysis.

**FIG 6:**
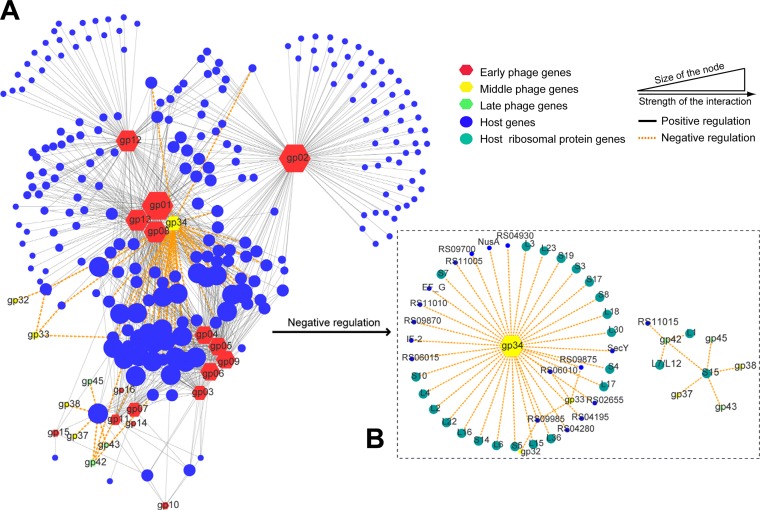
Gene coexpression network between φAbp1 and the host. (A) Main networks of host interactions centered on phage genes; (B) negative regulation network of *gp34*. The hexagons represent phage φAbp1 genes, marked with three colors of nodes, and circles indicate the host genes. The size of the nodes shows the interaction strength.

To screen genes with vital roles in the regulation of host gene expression, φAbp1-AB1 interaction networks based on gene coexpression analysis were constructed. First, we classified φAbp1 genes into early (*gp01* to *gp21* [*gp01-21*]), middle (*gp22-34*), and late (*gp35-54*) genes according to the gene clustering in Fig. 3 and the sequential expression of the φAbp1 phage gene. A total of 944 coexpression relationships between 240 host genes and 24 phage genes were screened by gene coexpression network analysis, including 49 negative correlations and 895 positive correlations. The results formed a network structure centered on the phage genes *gp01*, *gp08*, *gp13*, and *gp34*, indicating that these four genes may play a central role in interacting with the host genes (Fig. 6). Furthermore, the network also includes 3 subnetworks centered on *gp12*, *gp02*, and the *gp03-gp04-gp05-gp06-gp09* cluster. The expression patterns of *gp01*, *gp02*, *gp08*, *gp12*, and *gp34* were validated by real-time quantitative PCR (RT-qPCR) (Table S3, part I). Among these central genes, *gp34* was annotated as a phage-associated RNA polymerase, and other central phage genes were all early phage genes with unknown annotations (Table S4). Thus, in the negative regulation network, the results showed that *gp34* plays a core role.

10.1128/mSystems.00068-19.3 TABLE S3 Validation of host virulence and resistance genes and selected phage genes. Download Table S3, DOCX file, 0.02 MB. Copyright © 2019 Yang et al. 2019 Yang et al. This content is distributed under the terms of the Creative Commons Attribution 4.0 International license.

10.1128/mSystems.00068-19.4 TABLE S4 Phage-host interaction subnetworks between AB1 and φAbp1. Download Table S4, DOCX file, 0.01 MB. Copyright © 2019 Yang et al. 2019 Yang et al. This content is distributed under the terms of the Creative Commons Attribution 4.0 International license.

### Effects of φAbp1 infection on host resistance and virulence.

**FIG 7:**
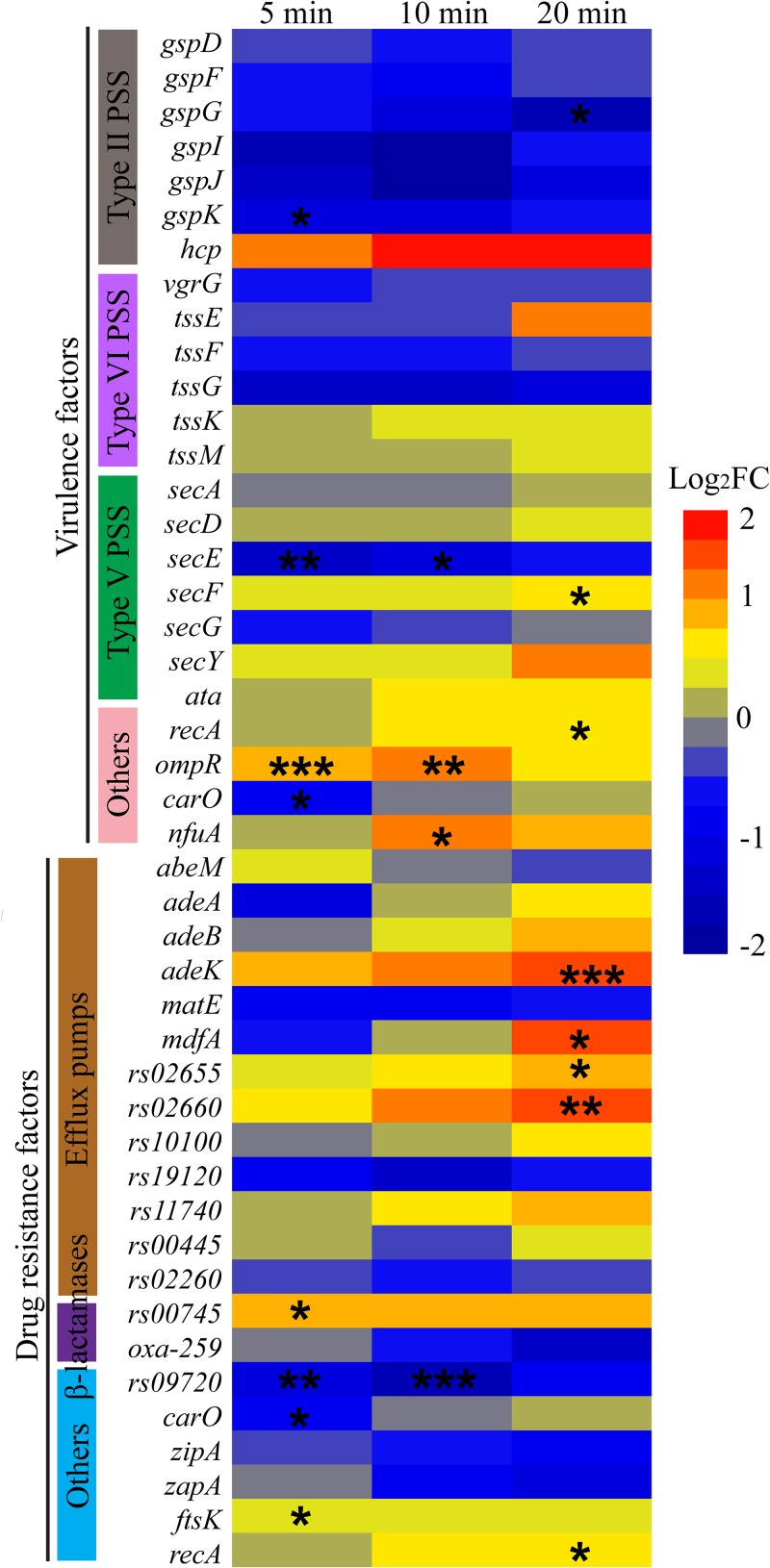
Expression of AB1 genes related to virulence and drug resistance after phage infection. The warm colors indicate upregulation, while the cold colors indicate downregulation. The asterisks indicate *q* values, where “*,” “**,” and “***” indicate *q* values of <0.05, <0.01, and <0.001, respectively. PSS indicates pili and secretion systems.

From a clinical perspective, it is important to consider the alterations in A. baumannii virulence and antibiotic resistance genes after φAbp1 infection. The expression of 24 virulence factors, including type II/V/VI secretory systems (*hcp* and *tssE*), porin (*carO*), and pili (*ompR*), and the expression of 21 resistance factors, including efflux pumps (*adeK*, *mdfA*, and *rs02660*) and beta-lactamases, were selected according to previous reports (24) and statistically analyzed by their differentially expressed patterns. The results showed that the expression of virulence factors was mainly inhibited after φAbp1 infection, but the drug resistance genes of the host were mainly activated (Fig. 7). The genes with significantly changed expression included 8 virulence factors and 9 resistance factors after φAbp1 infection (*P* < 0.05). It is noteworthy that 3 efflux pump-related genes appeared to be upregulated more than 1.5-fold at 20 min, suggesting that phage infection may alter the virulence and resistance of the host. All these host genes (resistance and virulence genes) were selected for further RT-qPCR validation. The validation result is consistent with the RNA-seq results (Table S3, part II). These genes should be paid attention in future studies of φAbp1 therapy and phage-derived antibacterial products.

## DISCUSSION

In this study, we progressed the transcriptional scheme of φAbp1 infection of AB1 and illustrated the interaction during φAbp1 predation in the host strain. Our results demonstrated that the expression of φAbp1 genes does not follow the standard lytic phage pattern. In the early infection stage after φAbp1 entered the cell (5 min), short and slight expression of the φAbp1 early genes was observed, with minimal impacts on the host. Next, during the middle stage (10 min), the expression of all φAbp1 middle genes peaked, as φAbp1 started to hijack cellular resources by regulating an increasing number of host DEGs. Finally, after most host cells were lysed, until phage-resistant mutants emerged and/or until lysogenic conversion occurred at the late stage of infection (80 min), a balance of phage-host interactions was achieved. Moreover, the majority of the host genes were constitutively expressed throughout the course of infection. However, upregulated host DEGs significantly overwhelmed downregulated DEGs during all stages of infection, indicating precise control by φAbp1 rather than simply forbidding host genes, as reported previously (19, 22, 25).

Phages take over host resources to propagate, including the manipulation of host proteins, molecular processes, cellular pathways that are related to transcription and translation, signal transduction, and metabolism (26). GO analysis in our work indicated that the host DEGs were regulated in a very precise way, including the irritation of stress reactions in the early infection stages. The GO analysis indicates a quick and thorough response from AB1 against φAbp1 infection, which has also been reported for the phage-host interaction between φR1-37 and Yersinia enterocolitica (23), and metabolic translation processes in the middle and late stages, indicating the assembly of phage particles and programmed downregulation of host material and nucleic acid biosynthetic/transfer processes. The upregulation of oxidation-reduction reactions and proteolysis by φAbp1 in its host indicates a rigorous defense response from AB1.

Moreover, clustering of significantly changed KEGG pathways revealed inhibited host nucleic acid complements, which was a consistent phenomenon in other phage infections (20, 27). However, significantly upregulated ribosome pathways and degradation or metabolism of multiple amino acid pathways are different from previous reports (22, 28). It was known that phage infection caused the shutoff of host macromolecular synthesis (DNA, RNA, and proteins) (28); however, the induction here might be for the synthesis of phage proteins in the middle stage of infection. Taking GO and KEGG results together, 10 min after infection was found to be the most remarkable time point when host nucleic acid-related biological processes were widely inhibited and amino acid-related pathways were extensively activated.

Phage early genes normally have been considered antibacterial candidates in a growing body of research (22). However, in this study, the φAbp1 early gene mainly showed positive regulations of host genes. Only the middle gene *gp34* was found to play a core role in the negative regulation network of ribosomal protein genes, suggesting that *gp34* may be a key gene that shuts off the host translation functions and that viral replication/transcription has surpassed that of the host cell, like previously reported T4 phage functions (29). Thus, *gp34* might be a key antibacterial agent. The late genes of φAbp1 encoding structural proteins were not observed in the network, suggesting the weak regulatory role of the phage late genes, as was also reported in our previous study (22).

GO and KEGG plus networking analyses together indicate a strong and precise phage control. Despite the rigorous challenge from the host stress response, the host material biosynthetic and transfer processes were all halted, while the reassembly of viral protein was promoted, which is consistent with other reports (30). These results suggest that the takeover and shutoff of host AB1 gene expression by φAbp1 was step-by-step rather than all of a sudden.

From the perspective of treating A. baumannii infections, it is crucial to know whether this remedy induced changes different from those of other antibiotics or whether this remedy induced changes in virulence and antibiotic resistance of A. baumannii. Previous reports have focused on transcriptomic changes when A. baumannii was treated with antibiotics, including imipenem (31, 32), colistin (33), doripenem (33), amikacin (31), and meropenem (31). The numbers of DEGs induced by antibiotics were 28 by amikacin (31), 417 by imipenem-meropenem (31) (88/68 in an earlier study [32]), and 400 by colistin (33). However, in our studies, the application of phage φAbp1 caused changes in significantly more DEGs (600/3,838; 15.6%) during the treatment process, affirming thoroughly and elaboratively controlled predation and killing by φAbp1 in AB1 infection rather than just a simple lysing action.

In addition to spreading virulence and antibiotic resistance markers among bacteria, bacteriophages were also proven to promote the expression and/or induction of virulence/resistance traits in infected cells (34) of Enterococcus faecalis (35), Pseudomonas aeruginosa (36), Bacillus anthracis (21), and Escherichia coli (37). All these examples correspond to prophages, while there was practically no research regarding the effect of lytic predation on host virulence and antibiotic resistance. In our study, we have proven that φAbp1 lytic predation promoted a general inhibition of the expression of virulence factors. However, the drug resistance genes of AB1 were partially activated after infection with φAbp1. The molecular mechanism of virulence/resistance alteration remains to be further studied. Previous papers all report good results from phage application, including excellent lysis ability and zero side effects (38–40). Our observation of activated drug resistance gene expression is a contraindication to previous studies, which might cause researchers to use caution in phage applications. Thus, this study may be a hint to study the influence of lytic phages on antibiotic resistance/virulence phenotypes in their hosts during treatment.

This transcriptomic study has its inherent shortcomings. For the whole phage predation and lysis process, we are still unable to elucidate the translation changes, especially when *gp34* significantly inhibited ribosome expression. Moreover, due to the lack of other validation experiments, some descriptions and discussions remain to be further studied.

In conclusion, we provide a general description of a global phage-host transcriptome interaction that grounds further research aimed at elucidating the indicated interactions between φAbp1 and XDR-AB1. Despite previous similar observations (22), we discovered new stage-dependent inhibition of host genes by φAbp1, a novel ribosome-centered *gp34* negative control, and partially elevated antibiotic resistance expression during lytic infection. Overall, in-depth analysis of the mechanism of host gene expression shutoff performed by the phage, as well as knowledge of the precise control by φAbp1, is pivotal for research on novel antibacterial compounds and the development for phage therapy.

## MATERIALS AND METHODS

### Bacterium preparation, antibiotic susceptibility testing, and phage preparation.

AB1 was isolated from a burn patient in the burn ward of Southwest Hospital during routine bacteria monitoring. The susceptibility of AB1 to various antibiotics (listed in Table S1 in the supplemental material) (Oxoid, Hampshire, UK) was determined by the Kirby-Bauer (KB) method according to previously reported procedures (4). The MIC assay was conducted with the Vitek 2 compact automated ID/AST instrument system (bioMérieux, Craponne, France) according to the manufacturer’s procedures (41). The antibiotic susceptibility results were interpreted according to Clinical and Laboratory Standards Institute criteria (42).

φAbp1 was previously screened against AB1 from hospital sewage at the sewage management center of Southwest Hospital (12). φAbp1 particles were collected and purified using the CsCl gradient ultracentrifugation method (9). Both AB1 and φAbp1 were stored in our laboratory at −80°C in glycerol. AB1 was inoculated aerobically at a 1:100 dilution in Luria-Bertani (LB) medium (Oxoid, Hampshire, UK) at 37°C overnight before the study.

### One-step growth curve.

Phage multiplication in the host can be divided into five processes: (i) attachment, (ii) penetration, (iii) biosynthesis, (iv) maturation, and (v) assembly and release. To determine the phage infection stage for sampling, a one-step growth curve of φAbp1 was measured as previously reported (9). Briefly, for one-step growth experiments, AB1 cells were infected with φAbp1 (MOI [multiplicity of infection] of 0.1) after a 5-min adsorption and centrifuged for 30 s at 13,000 × *g*. Unadsorbed phage was removed from the supernatant by washing twice with LB medium. The infected bacterial pellets were then resuspended in 5 ml LB medium, and the cultures were grown at 37°C with shaking at 160 rpm. Samples were taken at 5- or 10-min intervals (up to 80 min). The number of φAbp1 particles was immediately determined using the double-layer agar plaque method. Experiments were carried out in triplicate.

### Total RNA extraction.

A total volume of 10 ml AB1 culture (optical density at 600 nm [OD_600_] of 0.6) was infected with phage φAbp1 at an MOI of 10, while an equal volume of an uninfected AB1 culture served as the negative control. Six samples for RNA isolation were taken (1 ml) from the noninfected/infected culture at three time points postinfection (5, 10, and 20 min), with three biological duplications. RNA extraction was performed using the SV total RNA isolation system (Promega, Madison, WI, USA). RNA quantity and quality checks were performed using a Bioanalyzer (Agilent, Santa Clara, CA, USA) and the RNA 6000 Nano kit (Agilent, Santa Clara, CA, USA).

### RNA sequencing.

For RNA sequencing, total RNA from all 18 samples was depleted of rRNA using the Ribo-Zero rRNA removal kit for Gram-negative bacteria (Epicentre, Madison, WI, USA). The cDNA libraries were constructed and sequenced on an Illumina HiSeq 2500 sequencer (Illumina, San Diego, CA, USA), using paired-end 2- by 150-bp reads. The raw data and processed bam files were deposited in the Gene Expression Omnibus (GEO) database.

### Bioinformatic analyses.

RNA sequence reads were aligned to the A. baumannii strain ATCC 17978 (GenBank accession no. NZ_CP018664.1) and *Acinetobacter* phage φAbp1 (GenBank accession no. NC_021316.1) sequences using Bowtie2 (http://bowtie-bio.sourceforge.net/bowtie2/index.shtml). RNA-seq data analysis was performed with Tophat (http://ccb.jhu.edu/software/tophat/index.shtml) and Cufflinks (https://cole-trapnell-lab.github.io/cufflinks/cuffdiff/index.html). Gene expression values were determined by the expected number of fragments per FPKM and the false discovery rate (FDR) (*q* value). DESeq was used to calculate differentially expressed genes (DEGs) between the two groups. Genes with a fold change value (FC) of ≥1.5 and a *q* value of ≤0.05 were considered to be DEGs. GO (gene ontology) term enrichment of DEGs was performed with Blast2GO software (BioBam) based on Wallenius’ noncentral hypergeometric distribution. KOBAS (http://kobas.cbi.pku.edu.cn/) was used for KEGG pathway analysis. The analysis and visualization of gene coexpression networks were achieved by using Cytoscape 3.4.0 (https://cytoscape.org/).

### RT-qPCR validation of RNA-seq results.

Real-time quantitative PCR (RT-qPCR) analysis was further performed to validate the RNA-seq results. Seven AB1 (virulence- or drug resistance-related) genes and five φAbp1 (core regulatory) genes were selected for RT-qPCR validation. RT-qPCR was performed using SYBR Premix Ex *Taq* II (TaKaRa Bio, Dalian, China). The primers used in this study are listed in Table S5 in the supplemental material. The 16S rRNA gene was selected as the reference gene for normalization.

10.1128/mSystems.00068-19.5 TABLE S5 Primers used for RT-qPCR validation. Download Table S5, DOCX file, 0.02 MB. Copyright © 2019 Yang et al. 2019 Yang et al. This content is distributed under the terms of the Creative Commons Attribution 4.0 International license.

### Data availability.

The raw data and processed files were deposited in the NCBI GEO database with accession no. GSE117396.
