# Supplementary material for: Global Transcriptomic Analysis of the Interactions between Phage φAbp1 and Extensively Drug-Resistant Acinetobacter baumannii
Source: mSystems. 2019 Apr 16;4(2):e00068-19. doi: 10.1128/mSystems.00068-19 (PMC6469957; doi:10.1128/mSystems.00068-19)
Supplement: TABLE S1 [file mSystems.00068-19-st001.docx]

| Table S1 The antibiotic susceptibility results of *A.baumannii* AB1 | | | | | | |
| --- | --- | --- | --- | --- | --- | --- |
| Antibiotics | Drug resistance | Diameter  /Result | S | I | R | Measure |
| Piperacillin | Resistant | 6 | ≥21 | 18-20 | ≤17 | KB |
| Cefoperazone | Resistant | 11 | ≥22 | 15-21 | ≤14 |  |
| Ampicillin^*^ | Resistant | 8 | ≥15 | 12-14 | ≤11 |  |
| Piperacillin | Resistant | 6 | ≥21 | 18-20 | ≤17 |  |
| Ceftazidime | Resistant | 7 | ≥18 | 15 - 17 | ≤14 |  |
| Cefotaxime^*^ | Resistant | 6 | ≥18 | 15-22 | ≤14 |  |
| Cefepime | Resistant | 6 | ≥18 | 15 - 17 | ≤14 |  |
| Compound sulfamethoxazole | Resistant | 6 | ≥16 | 13-15 | ≤12 |  |
| Imipenem | Resistant | 7 | ≥22 | 19-21 | ≤18 |  |
| Tetracycline | Resistant | 6 | ≥15 | 12-14 | ≤11 |  |
| Meropenem | Resistant | 7 | ≥18 | 15 - 17 | ≤14 |  |
| Amikacin | Resistant | 7 | ≥14 | 15-16 | ≤17 |  |
| Gentamicin | Resistant | 6 | ≥15 | 13-14 | ≤12 |  |
| Tobramycin | Resistant | 6 | ≥15 | 13-14 | ≤12 |  |
| Ciprofloxacin | Resistant | 6 | ≥21 | 16-20 | ≤15 |  |
| Levofloxacin | Resistant | 7 | ≥17 | 14-16 | ≤13 |  |
| Minocycline | Resistant | 6 | ≥16 | 13-15 | ≤12 |  |
| Netilmicin | Resistant | 33 | ≤8ug/ml | 16 | ≥32ug/ml | MIC |
| Tigecycline | Intermediate | 1.5 | ≤1ug/ml | - | ≥4ug/ml |  |
| Minocycline | Resistant | 17.3 | ≤4 | 8 | ≥16 |  |
| Polymyxin B | Susceptible | 0.236 | ≤2ug/ml | - | ≥4ug/ml |  |

* indicates intrinsic resistance of A. baumannii.
