# Supplementary material for: Global Transcriptomic Analysis of the Interactions between Phage φAbp1 and Extensively Drug-Resistant Acinetobacter baumannii
Source: mSystems. 2019 Apr 16;4(2):e00068-19. doi: 10.1128/mSystems.00068-19 (PMC6469957; doi:10.1128/mSystems.00068-19)
Supplement: TABLE S2 [file mSystems.00068-19-st002.docx]

Table S2 Detailed GO expression data on specific genes of *A. baumannii* AB1

| GO_accession | Description | Group | Gene_list | Fold_  change | *q* Value |
| --- | --- | --- | --- | --- | --- |
| GO:0008150 | biological_process | 20min_Up | AUO97_RS11940 | 3.6809 | 0.03721 |
| GO:0055114 | oxidation-reduction process | 05min_Up | AUO97_RS10770 | 3.4043 | 3.99E-21 |
| GO:0008150 | biological_process | 20min_Up | AUO97_RS17615 | 3.3288 | 8.19E-12 |
| GO:0008150 | biological_process | 10min_Up | AUO97_RS10770 | 3.3284 | 6.44E-09 |
| GO:0008152 | metabolic process | 10min_Up | AUO97_RS10770 | 3.3284 | 6.44E-09 |
| GO:0055114 | oxidation-reduction process | 10min_Up | AUO97_RS10770 | 3.3284 | 6.44E-09 |
| GO:0008150 | biological_process | 10min_Up | AUO97_RS17615 | 3.1748 | 1.16E-12 |
| GO:0008150 | biological_process | 20min_Up | AUO97_RS14385 | 3.1521 | 2.93E-09 |
| GO:0008150 | biological_process | 10min_Up | AUO97_RS14385 | 3.0836 | 1.77E-13 |
| GO:0008152 | metabolic process | 10min_Up | AUO97_RS14385 | 3.0836 | 1.77E-13 |
| GO:0008150 | biological_process | 20min_Up | AUO97_RS11855 | 3.0463 | 2.17E-07 |
| GO:0008150 | biological_process | 10min_Up | AUO97_RS07600 | 2.9203 | 2.10E-05 |
| GO:0008150 | biological_process | 10min_Up | AUO97_RS12360 | 2.899 | 6.33E-07 |
| GO:0008152 | metabolic process | 10min_Up | AUO97_RS12360 | 2.899 | 6.33E-07 |
| GO:0055114 | oxidation-reduction process | 10min_Up | AUO97_RS12360 | 2.899 | 6.33E-07 |
| GO:0008150 | biological_process | 20min_Up | AUO97_RS18410 | 2.7575 | 4.12E-10 |
| GO:0008150 | biological_process | 20min_Up | AUO97_RS12360 | 2.7043 | 0.00203 |
| GO:0008150 | biological_process | 20min_Up | AUO97_RS14155 | 2.7005 | 0.03937 |
| GO:0008150 | biological_process | 20min_Up | AUO97_RS16510 | 2.6873 | 0.03157 |
| GO:0055114 | oxidation-reduction process | 05min_Up | AUO97_RS15920 | 2.6852 | 5.18E-09 |
| GO:0008150 | biological_process | 20min_Up | AUO97_RS18540 | 2.6046 | 0.0383 |
| GO:0008150 | biological_process | 20min_Up | AUO97_RS01630 | 2.5775 | 3.00E-06 |
| GO:0055114 | oxidation-reduction process | 05min_Up | AUO97_RS04560 | 2.5538 | 1.59E-19 |
| GO:0008150 | biological_process | 20min_Up | AUO97_RS04560 | 2.5513 | 3.00E-09 |
| GO:0008150 | biological_process | 10min_Up | AUO97_RS01630 | 2.5143 | 8.84E-08 |
| GO:0008152 | metabolic process | 10min_Up | AUO97_RS01630 | 2.5143 | 8.84E-08 |
| GO:0008150 | biological_process | 20min_Up | AUO97_RS14725 | 2.499 | 0.00018 |
| GO:0055114 | oxidation-reduction process | 05min_Up | AUO97_RS15925 | 2.4984 | 0.00313 |
| GO:0008150 | biological_process | 10min_Up | AUO97_RS14725 | 2.4686 | 1.48E-06 |
| GO:0008150 | biological_process | 10min_Up | AUO97_RS18410 | 2.4509 | 9.71E-06 |
| GO:0008152 | metabolic process | 10min_Up | AUO97_RS18410 | 2.4509 | 9.71E-06 |
| GO:0008150 | biological_process | 10min_Up | AUO97_RS11855 | 2.413 | 0.03593 |
| GO:0008152 | metabolic process | 10min_Up | AUO97_RS11855 | 2.413 | 0.03593 |
| GO:0055114 | oxidation-reduction process | 05min_Up | AUO97_RS18405 | 2.3948 | 3.75E-18 |
| GO:0006412 | translation | 20min_Up | AUO97_RS09225 | 2.3773 | 2.59E-08 |
| GO:0008150 | biological_process | 20min_Up | AUO97_RS09225 | 2.3773 | 2.59E-08 |
| GO:0008150 | biological_process | 10min_Up | AUO97_RS18690 | 2.3769 | 8.41E-07 |
| GO:0008152 | metabolic process | 10min_Up | AUO97_RS18690 | 2.3769 | 8.41E-07 |
| GO:0008150 | biological_process | 10min_Up | AUO97_RS07665 | 2.3151 | 0.03774 |
| GO:0008152 | metabolic process | 10min_Up | AUO97_RS07665 | 2.3151 | 0.03774 |
| GO:0008150 | biological_process | 10min_Up | AUO97_RS04560 | 2.3005 | 1.48E-06 |
| GO:0008152 | metabolic process | 10min_Up | AUO97_RS04560 | 2.3005 | 1.48E-06 |
| GO:0055114 | oxidation-reduction process | 10min_Up | AUO97_RS04560 | 2.3005 | 1.48E-06 |
| GO:0008150 | biological_process | 10min_Up | AUO97_RS16475 | 2.2823 | 0.04039 |
| GO:0008152 | metabolic process | 10min_Up | AUO97_RS16475 | 2.2823 | 0.04039 |
| GO:0006412 | translation | 20min_Up | AUO97_RS00545 | 2.2501 | 1.57E-08 |
| GO:0008150 | biological_process | 20min_Up | AUO97_RS00545 | 2.2501 | 1.57E-08 |
| GO:0008150 | biological_process | 10min_Up | AUO97_RS03195 | 2.2104 | 1.11E-07 |
| GO:0008150 | biological_process | 20min_Up | AUO97_RS13480 | 2.206 | 5.81E-05 |
| GO:0008150 | biological_process | 20min_Up | AUO97_RS18690 | 2.2056 | 1.32E-06 |
| GO:0008150 | biological_process | 10min_Up | AUO97_RS15920 | 2.1995 | 0.04153 |
| GO:0008152 | metabolic process | 10min_Up | AUO97_RS15920 | 2.1995 | 0.04153 |
| GO:0055114 | oxidation-reduction process | 10min_Up | AUO97_RS15920 | 2.1995 | 0.04153 |
| GO:0008150 | biological_process | 10min_Up | AUO97_RS03160 | 2.1345 | 6.22E-07 |
| GO:0008150 | biological_process | 20min_Up | AUO97_RS02205 | 2.1122 | 0.02802 |
| GO:0008150 | biological_process | 20min_Up | AUO97_RS11860 | 2.1086 | 0.04243 |
| GO:0008150 | biological_process | 10min_Up | AUO97_RS18540 | 2.0879 | 0.01046 |
| GO:0008152 | metabolic process | 10min_Up | AUO97_RS18540 | 2.0879 | 0.01046 |
| GO:0008150 | biological_process | 10min_Up | AUO97_RS14095 | 2.0782 | 2.18E-05 |
| GO:0055114 | oxidation-reduction process | 05min_Up | AUO97_RS12360 | 2.0755 | 0.00017 |
| GO:0008150 | biological_process | 10min_Up | AUO97_RS11860 | 2.0692 | 0.01979 |
| GO:0008150 | biological_process | 20min_Up | AUO97_RS04640 | 2.0531 | 0.00262 |
| GO:0008150 | biological_process | 10min_Up | AUO97_RS18405 | 2.0419 | 3.83E-06 |
| GO:0008152 | metabolic process | 10min_Up | AUO97_RS18405 | 2.0419 | 3.83E-06 |
| GO:0055114 | oxidation-reduction process | 10min_Up | AUO97_RS18405 | 2.0419 | 3.83E-06 |
| GO:0008150 | biological_process | 20min_Up | AUO97_RS03195 | 2.0393 | 6.67E-07 |
| GO:0008150 | biological_process | 20min_Up | AUO97_RS10295 | 2.0358 | 0.0063 |
| GO:0008150 | biological_process | 10min_Up | AUO97_RS15925 | 2.0237 | 0.01357 |
| GO:0008152 | metabolic process | 10min_Up | AUO97_RS15925 | 2.0237 | 0.01357 |
| GO:0055114 | oxidation-reduction process | 10min_Up | AUO97_RS15925 | 2.0237 | 0.01357 |
| GO:0008150 | biological_process | 20min_Up | AUO97_RS14735 | 2.0168 | 0.01146 |
| GO:0008150 | biological_process | 10min_Up | AUO97_RS18975 | 2.0141 | 2.33E-06 |
| GO:0008152 | metabolic process | 10min_Up | AUO97_RS18975 | 2.0141 | 2.33E-06 |
| GO:0055114 | oxidation-reduction process | 10min_Up | AUO97_RS18975 | 2.0141 | 2.33E-06 |
| GO:0008150 | biological_process | 10min_Up | AUO97_RS04270 | 1.9949 | 2.72E-05 |
| GO:0008152 | metabolic process | 10min_Up | AUO97_RS04270 | 1.9949 | 2.72E-05 |
| GO:0055114 | oxidation-reduction process | 10min_Up | AUO97_RS04270 | 1.9949 | 2.72E-05 |
| GO:0008150 | biological_process | 10min_Up | AUO97_RS04725 | 1.9781 | 3.34E-06 |
| GO:0008152 | metabolic process | 10min_Up | AUO97_RS04725 | 1.9781 | 3.34E-06 |
| GO:0055114 | oxidation-reduction process | 10min_Up | AUO97_RS04725 | 1.9781 | 3.34E-06 |
| GO:0008150 | biological_process | 20min_Up | AUO97_RS18975 | 1.9707 | 0.00297 |
| GO:0008150 | biological_process | 10min_Up | AUO97_RS02840 | 1.9608 | 0.04677 |
| GO:0008152 | metabolic process | 10min_Up | AUO97_RS02840 | 1.9608 | 0.04677 |
| GO:0055114 | oxidation-reduction process | 10min_Up | AUO97_RS02840 | 1.9608 | 0.04677 |
| GO:0006790 | sulfur compound metabolic process | 10min_Up | AUO97_RS07375 | 1.959 | 1.62E-05 |
| GO:0008150 | biological_process | 10min_Up | AUO97_RS07375 | 1.959 | 1.62E-05 |
| GO:0008152 | metabolic process | 10min_Up | AUO97_RS07375 | 1.959 | 1.62E-05 |
| GO:0008150 | biological_process | 20min_Up | AUO97_RS18405 | 1.9543 | 2.75E-05 |
| GO:0008150 | biological_process | 10min_Up | AUO97_RS14995 | 1.9398 | 0.00012 |
| GO:0008152 | metabolic process | 10min_Up | AUO97_RS14995 | 1.9398 | 0.00012 |
| GO:0008150 | biological_process | 10min_Up | AUO97_RS07040 | 1.9348 | 5.40E-06 |
| GO:0008152 | metabolic process | 10min_Up | AUO97_RS07040 | 1.9348 | 5.40E-06 |
| GO:0055114 | oxidation-reduction process | 10min_Up | AUO97_RS07040 | 1.9348 | 5.40E-06 |
| GO:0055114 | oxidation-reduction process | 05min_Up | AUO97_RS04270 | 1.9273 | 3.05E-10 |
| GO:0008150 | biological_process | 10min_Up | AUO97_RS09225 | 1.9115 | 4.94E-06 |
| GO:0008152 | metabolic process | 10min_Up | AUO97_RS09225 | 1.9115 | 4.94E-06 |
| GO:0008150 | biological_process | 10min_Up | AUO97_RS12965 | 1.8934 | 7.23E-06 |
| GO:0008152 | metabolic process | 10min_Up | AUO97_RS12965 | 1.8934 | 7.23E-06 |
| GO:0009057 | macromolecule catabolic process | 10min_Up | AUO97_RS12965 | 1.8934 | 7.23E-06 |
| GO:0044265 | cellular macromolecule catabolic process | 10min_Up | AUO97_RS12965 | 1.8934 | 7.23E-06 |
| GO:0008150 | biological_process | 20min_Up | AUO97_RS04270 | 1.8932 | 0.00012 |
| GO:0008150 | biological_process | 10min_Up | AUO97_RS19115 | 1.8913 | 2.64E-05 |
| GO:0008152 | metabolic process | 10min_Up | AUO97_RS19115 | 1.8913 | 2.64E-05 |
| GO:0008150 | biological_process | 10min_Up | AUO97_RS11015 | 1.89 | 0.04353 |
| GO:0008152 | metabolic process | 10min_Up | AUO97_RS11015 | 1.89 | 0.04353 |
| GO:0055114 | oxidation-reduction process | 10min_Up | AUO97_RS11015 | 1.89 | 0.04353 |
| GO:0008150 | biological_process | 10min_Up | AUO97_RS10220 | 1.8866 | 9.45E-06 |
| GO:0008152 | metabolic process | 10min_Up | AUO97_RS10220 | 1.8866 | 9.45E-06 |
| GO:0008150 | biological_process | 20min_Up | AUO97_RS02375 | 1.8721 | 3.38E-05 |
| GO:0008150 | biological_process | 10min_Up | AUO97_RS07850 | 1.8431 | 0.00524 |
| GO:0008152 | metabolic process | 10min_Up | AUO97_RS07850 | 1.8431 | 0.00524 |
| GO:0008150 | biological_process | 10min_Up | AUO97_RS10295 | 1.815 | 0.00412 |
| GO:0008152 | metabolic process | 10min_Up | AUO97_RS10295 | 1.815 | 0.00412 |
| GO:0008150 | biological_process | 20min_Up | AUO97_RS07870 | 1.7859 | 0.00265 |
| GO:0008150 | biological_process | 10min_Up | AUO97_RS03140 | 1.7756 | 0.00828 |
| GO:0008152 | metabolic process | 10min_Up | AUO97_RS03140 | 1.7756 | 0.00828 |
| GO:0008150 | biological_process | 20min_Up | AUO97_RS11745 | 1.7679 | 0.01308 |
| GO:0008150 | biological_process | 10min_Up | AUO97_RS13880 | 1.7583 | 0.00011 |
| GO:0008152 | metabolic process | 10min_Up | AUO97_RS13880 | 1.7583 | 0.00011 |
| GO:0055114 | oxidation-reduction process | 10min_Up | AUO97_RS13880 | 1.7583 | 0.00011 |
| GO:0008150 | biological_process | 20min_Up | AUO97_RS07375 | 1.7564 | 0.00201 |
| GO:0008150 | biological_process | 20min_Up | AUO97_RS14995 | 1.744 | 0.00301 |
| GO:0006508 | proteolysis | 10min_Up | AUO97_RS17380 | 1.742 | 4.96E-05 |
| GO:0008150 | biological_process | 10min_Up | AUO97_RS17380 | 1.742 | 4.96E-05 |
| GO:0008152 | metabolic process | 10min_Up | AUO97_RS17380 | 1.742 | 4.96E-05 |
| GO:0008150 | biological_process | 10min_Up | AUO97_RS17010 | 1.739 | 0.00197 |
| GO:0008152 | metabolic process | 10min_Up | AUO97_RS17010 | 1.739 | 0.00197 |
| GO:0055114 | oxidation-reduction process | 10min_Up | AUO97_RS17010 | 1.739 | 0.00197 |
| GO:0008150 | biological_process | 20min_Up | AUO97_RS02660 | 1.7371 | 0.00767 |
| GO:0008150 | biological_process | 10min_Up | AUO97_RS14970 | 1.736 | 5.43E-05 |
| GO:0008150 | biological_process | 20min_Up | AUO97_RS17380 | 1.7249 | 5.82E-05 |
| GO:0008150 | biological_process | 10min_Up | AUO97_RS11220 | 1.7248 | 0.00014 |
| GO:0008152 | metabolic process | 10min_Up | AUO97_RS11220 | 1.7248 | 0.00014 |
| GO:0055114 | oxidation-reduction process | 05min_Up | AUO97_RS13880 | 1.7212 | 5.76E-09 |
| GO:0008150 | biological_process | 10min_Up | AUO97_RS02205 | 1.719 | 0.01881 |
| GO:0008152 | metabolic process | 10min_Up | AUO97_RS02205 | 1.719 | 0.01881 |
| GO:0008150 | biological_process | 20min_Up | AUO97_RS09120 | 1.718 | 0.04567 |
| GO:0008150 | biological_process | 20min_Up | AUO97_RS14740 | 1.713 | 0.01842 |
| GO:0055114 | oxidation-reduction process | 05min_Up | AUO97_RS14380 | 1.712 | 6.41E-09 |
| GO:0008150 | biological_process | 10min_Up | AUO97_RS09380 | 1.7092 | 0.01239 |
| GO:0008152 | metabolic process | 10min_Up | AUO97_RS09380 | 1.7092 | 0.01239 |
| GO:0008150 | biological_process | 10min_Up | AUO97_RS02375 | 1.6966 | 0.00011 |
| GO:0008152 | metabolic process | 10min_Up | AUO97_RS02375 | 1.6966 | 0.00011 |
| GO:0008150 | biological_process | 20min_Up | AUO97_RS05440 | 1.6959 | 0.00113 |
| GO:0008150 | biological_process | 20min_Up | AUO97_RS14115 | 1.6954 | 0.02299 |
| GO:0008150 | biological_process | 20min_Up | AUO97_RS12965 | 1.6815 | 0.00022 |
| GO:0008150 | biological_process | 10min_Up | AUO97_RS05440 | 1.6809 | 0.00028 |
| GO:0008152 | metabolic process | 10min_Up | AUO97_RS05440 | 1.6809 | 0.00028 |
| GO:0008150 | biological_process | 20min_Up | AUO97_RS01255 | 1.6778 | 0.00137 |
| GO:0008150 | biological_process | 20min_Up | AUO97_RS18965 | 1.6711 | 1.82E-05 |
| GO:0009060 | aerobic respiration | 20min_Up | AUO97_RS18965 | 1.6711 | 1.82E-05 |
| GO:0015980 | energy derivation by oxidation of organic compounds | 20min_Up | AUO97_RS18965 | 1.6711 | 1.82E-05 |
| GO:0045333 | cellular respiration | 20min_Up | AUO97_RS18965 | 1.6711 | 1.82E-05 |
| GO:0008150 | biological_process | 10min_Up | AUO97_RS10455 | 1.6637 | 0.00315 |
| GO:0008152 | metabolic process | 10min_Up | AUO97_RS10455 | 1.6637 | 0.00315 |
| GO:0008150 | biological_process | 20min_Up | AUO97_RS07040 | 1.639 | 0.00012 |
| GO:0008150 | biological_process | 20min_Up | AUO97_RS11220 | 1.6385 | 0.0002 |
| GO:0008150 | biological_process | 20min_Up | AUO97_RS10220 | 1.6278 | 0.00013 |
| GO:0006790 | sulfur compound metabolic process | 10min_Up | AUO97_RS08990 | 1.5857 | 0.0201 |
| GO:0008150 | biological_process | 10min_Up | AUO97_RS08990 | 1.5857 | 0.0201 |
| GO:0008152 | metabolic process | 10min_Up | AUO97_RS08990 | 1.5857 | 0.0201 |
| GO:0008150 | biological_process | 10min_Up | AUO97_RS18965 | 1.5856 | 0.03082 |
| GO:0008152 | metabolic process | 10min_Up | AUO97_RS18965 | 1.5856 | 0.03082 |
| GO:0055114 | oxidation-reduction process | 10min_Up | AUO97_RS18965 | 1.5856 | 0.03082 |
| GO:0008150 | biological_process | 20min_Up | AUO97_RS11015 | 1.5829 | 0.01291 |
| GO:0015980 | energy derivation by oxidation of organic compounds | 20min_Up | AUO97_RS11015 | 1.5829 | 0.01291 |
| GO:0045333 | cellular respiration | 20min_Up | AUO97_RS11015 | 1.5829 | 0.01291 |
| GO:0006790 | sulfur compound metabolic process | 10min_Up | AUO97_RS15875 | 1.5827 | 0.00083 |
| GO:0008150 | biological_process | 10min_Up | AUO97_RS15875 | 1.5827 | 0.00083 |
| GO:0008152 | metabolic process | 10min_Up | AUO97_RS15875 | 1.5827 | 0.00083 |
| GO:0008150 | biological_process | 20min_Up | AUO97_RS11320 | 1.5733 | 0.04622 |
| GO:0006412 | translation | 20min_Up | AUO97_RS11530 | 1.5698 | 0.00941 |
| GO:0008150 | biological_process | 20min_Up | AUO97_RS11530 | 1.5698 | 0.00941 |
| GO:0008150 | biological_process | 10min_Up | AUO97_RS07870 | 1.5678 | 0.0019 |
| GO:0008152 | metabolic process | 10min_Up | AUO97_RS07870 | 1.5678 | 0.0019 |
| GO:0055114 | oxidation-reduction process | 10min_Up | AUO97_RS07870 | 1.5678 | 0.0019 |
| GO:0008150 | biological_process | 20min_Up | AUO97_RS07600 | 1.5544 | 0.01894 |
| GO:0008150 | biological_process | 20min_Up | AUO97_RS04725 | 1.549 | 0.00075 |
| GO:0055114 | oxidation-reduction process | 05min_Up | AUO97_RS04725 | 1.546 | 3.03E-07 |
| GO:0006412 | translation | 20min_Up | AUO97_RS08855 | 1.5439 | 7.24E-05 |
| GO:0006417 | regulation of translation | 20min_Up | AUO97_RS08855 | 1.5439 | 7.24E-05 |
| GO:0008150 | biological_process | 20min_Up | AUO97_RS08855 | 1.5439 | 7.24E-05 |
| GO:0010608 | posttranscriptional regulation of gene expression | 20min_Up | AUO97_RS08855 | 1.5439 | 7.24E-05 |
| GO:0032268 | regulation of cellular protein metabolic process | 20min_Up | AUO97_RS08855 | 1.5439 | 7.24E-05 |
| GO:0034248 | regulation of cellular amide metabolic process | 20min_Up | AUO97_RS08855 | 1.5439 | 7.24E-05 |
| GO:0051246 | regulation of protein metabolic process | 20min_Up | AUO97_RS08855 | 1.5439 | 7.24E-05 |
| GO:0008150 | biological_process | 20min_Up | AUO97_RS11840 | 1.5353 | 0.00039 |
| GO:0009060 | aerobic respiration | 20min_Up | AUO97_RS11840 | 1.5353 | 0.00039 |
| GO:0015980 | energy derivation by oxidation of organic compounds | 20min_Up | AUO97_RS11840 | 1.5353 | 0.00039 |
| GO:0045333 | cellular respiration | 20min_Up | AUO97_RS11840 | 1.5353 | 0.00039 |
| GO:0008150 | biological_process | 20min_Up | AUO97_RS09310 | 1.5337 | 0.00028 |
| GO:0055114 | oxidation-reduction process | 05min_Up | AUO97_RS12925 | 1.5274 | 0.0465 |
| GO:0008150 | biological_process | 10min_Up | AUO97_RS00545 | 1.5173 | 0.0357 |
| GO:0008152 | metabolic process | 10min_Up | AUO97_RS00545 | 1.5173 | 0.0357 |
| GO:0008150 | biological_process | 10min_Up | AUO97_RS10590 | 1.5154 | 0.00061 |
| GO:0008152 | metabolic process | 10min_Up | AUO97_RS10590 | 1.5154 | 0.00061 |
| GO:0016042 | lipid catabolic process | 05min_Up | AUO97_RS07850 | 1.5018 | 1.72E-05 |
| GO:0044281 | small molecule metabolic process | 10min_Down | AUO97_RS18650 | -1.5008 | 0.03319 |
| GO:0044283 | small molecule biosynthetic process | 05min_Down | AUO97_RS07775 | -1.5014 | 6.14E-06 |
| GO:0090407 | organophosphate biosynthetic process | 05min_Down | AUO97_RS07775 | -1.5014 | 6.14E-06 |
| GO:1901566 | organonitrogen compound biosynthetic process | 05min_Down | AUO97_RS07775 | -1.5014 | 6.14E-06 |
| GO:0065008 | regulation of biological quality | 10min_Down | AUO97_RS08670 | -1.5231 | 0.0004 |
| GO:1901135 | carbohydrate derivative metabolic process | 10min_Down | AUO97_RS08670 | -1.5231 | 0.0004 |
| GO:1901137 | carbohydrate derivative biosynthetic process | 10min_Down | AUO97_RS08670 | -1.5231 | 0.0004 |
| GO:0044283 | small molecule biosynthetic process | 05min_Down | AUO97_RS17945 | -1.533 | 0.00018 |
| GO:1901566 | organonitrogen compound biosynthetic process | 05min_Down | AUO97_RS17945 | -1.533 | 0.00018 |
| GO:0006857 | oligopeptide transport | 20min_Down | AUO97_RS00685 | -1.5376 | 0.00099 |
| GO:0044283 | small molecule biosynthetic process | 05min_Down | AUO97_RS13575 | -1.5446 | 1.25E-06 |
| GO:1901566 | organonitrogen compound biosynthetic process | 05min_Down | AUO97_RS13575 | -1.5446 | 1.25E-06 |
| GO:0006220 | pyrimidine nucleotide metabolic process | 10min_Down | AUO97_RS17760 | -1.583 | 5.64E-05 |
| GO:0006221 | pyrimidine nucleotide biosynthetic process | 10min_Down | AUO97_RS17760 | -1.583 | 5.64E-05 |
| GO:0006793 | phosphorus metabolic process | 10min_Down | AUO97_RS17760 | -1.583 | 5.64E-05 |
| GO:0044281 | small molecule metabolic process | 10min_Down | AUO97_RS17760 | -1.583 | 5.64E-05 |
| GO:0072527 | pyrimidine-containing compound metabolic process | 10min_Down | AUO97_RS17760 | -1.583 | 5.64E-05 |
| GO:0072528 | pyrimidine-containing compound biosynthetic process | 10min_Down | AUO97_RS17760 | -1.583 | 5.64E-05 |
| GO:0090407 | organophosphate biosynthetic process | 10min_Down | AUO97_RS17760 | -1.583 | 5.64E-05 |
| GO:1901135 | carbohydrate derivative metabolic process | 10min_Down | AUO97_RS17760 | -1.583 | 5.64E-05 |
| GO:1901137 | carbohydrate derivative biosynthetic process | 10min_Down | AUO97_RS17760 | -1.583 | 5.64E-05 |
| GO:0006793 | phosphorus metabolic process | 10min_Down | AUO97_RS09720 | -1.5898 | 0.00046 |
| GO:0044281 | small molecule metabolic process | 10min_Down | AUO97_RS09720 | -1.5898 | 0.00046 |
| GO:0065008 | regulation of biological quality | 10min_Down | AUO97_RS09720 | -1.5898 | 0.00046 |
| GO:0090407 | organophosphate biosynthetic process | 10min_Down | AUO97_RS09720 | -1.5898 | 0.00046 |
| GO:0065008 | regulation of biological quality | 10min_Down | AUO97_RS02890 | -1.5906 | 0.00439 |
| GO:0044281 | small molecule metabolic process | 10min_Down | AUO97_RS15215 | -1.6537 | 0.00069 |
| GO:0065008 | regulation of biological quality | 10min_Down | AUO97_RS15215 | -1.6537 | 0.00069 |
| GO:0006220 | pyrimidine nucleotide metabolic process | 10min_Down | AUO97_RS01540 | -1.666 | 0.04793 |
| GO:0006221 | pyrimidine nucleotide biosynthetic process | 10min_Down | AUO97_RS01540 | -1.666 | 0.04793 |
| GO:0006793 | phosphorus metabolic process | 10min_Down | AUO97_RS01540 | -1.666 | 0.04793 |
| GO:0044281 | small molecule metabolic process | 10min_Down | AUO97_RS01540 | -1.666 | 0.04793 |
| GO:0072527 | pyrimidine-containing compound metabolic process | 10min_Down | AUO97_RS01540 | -1.666 | 0.04793 |
| GO:0072528 | pyrimidine-containing compound biosynthetic process | 10min_Down | AUO97_RS01540 | -1.666 | 0.04793 |
| GO:0090407 | organophosphate biosynthetic process | 10min_Down | AUO97_RS01540 | -1.666 | 0.04793 |
| GO:1901135 | carbohydrate derivative metabolic process | 10min_Down | AUO97_RS01540 | -1.666 | 0.04793 |
| GO:1901137 | carbohydrate derivative biosynthetic process | 10min_Down | AUO97_RS01540 | -1.666 | 0.04793 |
| GO:0044281 | small molecule metabolic process | 10min_Down | AUO97_RS13575 | -1.6749 | 5.70E-05 |
| GO:0090407 | organophosphate biosynthetic process | 05min_Down | AUO97_RS18665 | -1.709 | 0.00155 |
| GO:1901137 | carbohydrate derivative biosynthetic process | 05min_Down | AUO97_RS18665 | -1.709 | 0.00155 |
| GO:0044281 | small molecule metabolic process | 10min_Down | AUO97_RS01895 | -1.7331 | 2.12E-05 |
| GO:0065008 | regulation of biological quality | 10min_Down | AUO97_RS17750 | -1.8048 | 4.62E-05 |
| GO:1901135 | carbohydrate derivative metabolic process | 10min_Down | AUO97_RS17750 | -1.8048 | 4.62E-05 |
| GO:1901137 | carbohydrate derivative biosynthetic process | 10min_Down | AUO97_RS17750 | -1.8048 | 4.62E-05 |
| GO:0000413 | protein peptidyl-prolyl isomerization | 20min_Down | AUO97_RS13840 | -1.8096 | 0.02802 |
| GO:0018208 | peptidyl-proline modification | 20min_Down | AUO97_RS13840 | -1.8096 | 0.02802 |
| GO:1901566 | organonitrogen compound biosynthetic process | 05min_Down | AUO97_RS11340 | -1.8245 | 3.05E-10 |
| GO:0034404 | nucleobase-containing small molecule biosynthetic process | 05min_Down | AUO97_RS09780 | -1.8276 | 0.00044 |
| GO:0044283 | small molecule biosynthetic process | 05min_Down | AUO97_RS09780 | -1.8276 | 0.00044 |
| GO:0006396 | RNA processing | 05min_Down | AUO97_RS01900 | -1.8348 | 3.26E-08 |
| GO:0006793 | phosphorus metabolic process | 10min_Down | AUO97_RS07775 | -2.0378 | 5.16E-05 |
| GO:0044281 | small molecule metabolic process | 10min_Down | AUO97_RS07775 | -2.0378 | 5.16E-05 |
| GO:0072527 | pyrimidine-containing compound metabolic process | 10min_Down | AUO97_RS07775 | -2.0378 | 5.16E-05 |
| GO:0072528 | pyrimidine-containing compound biosynthetic process | 10min_Down | AUO97_RS07775 | -2.0378 | 5.16E-05 |
| GO:0090407 | organophosphate biosynthetic process | 10min_Down | AUO97_RS07775 | -2.0378 | 5.16E-05 |
| GO:0006793 | phosphorus metabolic process | 10min_Down | AUO97_RS18665 | -2.1131 | 0.01444 |
| GO:0090407 | organophosphate biosynthetic process | 10min_Down | AUO97_RS18665 | -2.1131 | 0.01444 |
| GO:1901135 | carbohydrate derivative metabolic process | 10min_Down | AUO97_RS18665 | -2.1131 | 0.01444 |
| GO:1901137 | carbohydrate derivative biosynthetic process | 10min_Down | AUO97_RS18665 | -2.1131 | 0.01444 |
| GO:0044281 | small molecule metabolic process | 10min_Down | AUO97_RS07690 | -2.1743 | 0.02127 |
| GO:0090407 | organophosphate biosynthetic process | 05min_Down | AUO97_RS02275 | -3.1666 | 0.0002 |
