# Supplementary material for: Global Transcriptomic Analysis of the Interactions between Phage φAbp1 and Extensively Drug-Resistant Acinetobacter baumannii
Source: mSystems. 2019 Apr 16;4(2):e00068-19. doi: 10.1128/mSystems.00068-19 (PMC6469957; doi:10.1128/mSystems.00068-19)
Supplement: TABLE S3 [file mSystems.00068-19-st003.docx]

Table S3 validation of phage genes (Part I) and host virulence/resistance gene (Part II)

| Part I | | | | | | | | |
| --- | --- | --- | --- | --- | --- | --- | --- | --- |
| Phage  gene name | Readcounts in*  RNA-seq | | | | Relative expression level  RT-qPCR | | | |
|  | 5min | 10min | 20min | 5min | | | 10min | 20min |
| *gp01* | 19317.56 | 21661.26 | 15312.07 | 0.56 | | | 0.89 | 0.69 |
| *gp02* | 196840.7 | 163356.7 | 67409.66 | 0.60 | | | 0.23 | 0.02 |
| *gp08* | 15310.49 | 16027.08 | 394271.1 | 0.60 | | | 0.99 | 0.52 |
| *gp12* | 237.4614 | 262.2713 | 131800.2 | 0.12 | | | 0.11 | 0.13 |
| *gp34* | 21021.07 | 343444.9 | 125543.5 | 0.21 | | | 0.76 | 0.51 |
| Part II | | | | | | | | |
| Host  gene name | Fold change (q value) | | | | | | | |
|  | RNA-seq | | | | | RT-qPCR | | |
|  | 5min | 10min | 20min | | | 5min | 10min | 20min |
| *hcp* | 1.39(0.41) | 1.95(0.07) | 2.16(0.08) | | | 2.17 | 3.53 | 3.51 |
| *gspG* | -0.46(0.38) | -0.87(0.16) | -1.4(0.01) | | | -0.51 | -0.97 | -1.3 |
| *gspK* | -1.05(0.025) | -1.00(0.02) | -0.54(0.09) | | | -1.22 | -1.53 | -1.35 |
| *secE* | -1.22(<0.01) | -1.02(0.03) | -0.54(0.04) | | | -1.33 | -1.42 | -0.78 |
| *secF* | 0.63(0.55) | 0.79(0.23) | 0.94(0.03) | | | 0.77 | 0.91 | 1.13 |
| *ompR* | 1.10(<0.01) | 1.37(<0.01) | 0.84(0.13) | | | 1.32 | 1.51 | 1.01 |
| *nfuA* | 0.47(0.33) | 1.38(<0.01) | 1.28(0.55) | | | 0.51 | 1.41 | 1.33 |
| *adeK* | 1.08(0.21) | 1.48(0.07) | 1.66(<0.01) | | | 1.74 | 2.30 | 3.48 |
| *mdfA* | -0.42(0.30) | 0.32(0.74) | 1.69(0.03) | | | 0.57 | 1.23 | 2.46 |
| *RS02655* | 0.59(0.58) | 0.87(0.39) | 1.12(0.04) | | | 0.73 | 0.99 | 1.35 |
| *RS02660* | 0.97(0.32) | 1.42(0.19) | 1.74(<0.01) | | | 1.3 | 2.19 | 3.43 |
| *RS00745* | 1.21(0.02) | 1.18(0.27) | 1.08(<0.01) | | | 1.33 | 1.21 | 1.19 |
| *RS09720* | -0.99(<0.01) | -1.59(<0.01) | -0.78(0.38) | | | -1.01 | -1.89 | -1.01 |
| *carO* | -0.64(0.04) | 0.14(0.91) | 0.35(0.66) | | | 0.43 | 0.85 | 1.13 |
| *ftsK* | 0.77(0.02) | 0.58(0.29) | 0.73(0.14) | | | 0.87 | 0.63 | 0.83 |
| *recA* | 0.46(0.32) | 0.90(0.05) | 1.02(0.03) | | | 1.07 | 1.27 | 1.29 |

* Since the control group contains no phage genome, the readcounts of phage gene are presented in RNA-seq instead of fold change values. The relative expression of phage genes (against host 16S RNA) was calculated and presented in RT-qPCR.
