# Supplementary material for: Global Transcriptomic Analysis of the Interactions between Phage φAbp1 and Extensively Drug-Resistant Acinetobacter baumannii
Source: mSystems. 2019 Apr 16;4(2):e00068-19. doi: 10.1128/mSystems.00068-19 (PMC6469957; doi:10.1128/mSystems.00068-19)
Supplement: TABLE S4 [file mSystems.00068-19-st004.docx]

| Table S4 Phage-host interaction subnetworks between AB1 and φAbp1. | | |
| --- | --- | --- |
| Subnetwork | Phage gene | Annotation |
| subnetwork 1 | *gp01* | hypothetical protein |
|  | *gp08* | hypothetical protein |
|  | *gp13* | hypothetical protein |
|  | *gp34* | phage-associated RNA polymerase |
| subnetwork 2 | *gp12* | hypothetical protein |
| subnetwork 3 | *gp02* | hypothetical protein |
| subnetwork 4 | *gp03* | hypothetical protein |
|  | *gp04* | hypothetical protein |
|  | *gp05* | hypothetical protein |
|  | *gp06* | hypothetical protein |
|  | *gp09* | hypothetical protein |
