# Supplementary material for: Global Transcriptomic Analysis of the Interactions between Phage φAbp1 and Extensively Drug-Resistant Acinetobacter baumannii
Source: mSystems. 2019 Apr 16;4(2):e00068-19. doi: 10.1128/mSystems.00068-19 (PMC6469957; doi:10.1128/mSystems.00068-19)
Supplement: TABLE S5 [file mSystems.00068-19-st005.docx]

| Table S5 Primers used for RT-qPCR validation | | | |
| --- | --- | --- | --- |
| Gene name | Forward primer 5' to 3' | Reverse primer 5' to 3' | Product size |
| *16s* | CGGACGGGTGAGTAATGTCT | CTCAGACCAGCTAGGGATCG | 174 |
| *gp01* | CACTCTGATGGTAAGTTATGGGTTA | AAGCTGACGCACTTTTAACAGTATT | 154 |
| *gp02* | CGATATGTCGATGGAAGGTG | ATCAGCATCTAACGCTGCAA | 175 |
| *gp08* | GGAGGCTTTAATGGCTAACG | TGCTCATGTTCTTCCTTGTTAGG | 108 |
| *gp12* | AAAGGTTATAGTGTTGCACAAGC | ACCCACGCATTTCTTTTAGC | 155 |
| *gp34* | CTGAACCCTTAGCGAAGCAC | ACTAGCTCTGCGCCATTGTT | 223 |
| *hcp* | GGAAGCTTGTTCAGCTGGTT | CTGTAGGAACGCCTTCTTCG | 155 |
| *gspG* | GGCTTTACCCTTATTGAAGTTATG | GGAAATGCCCATTATCGAGC | 175 |
| *gspK* | GCAGAAGCCTTTTTTTCAGA | GCATCTTCATTTACAATACCTTC | 200 |
| *secE* | CTGCTGAAGTTGTTCGTTCT | GCCACAACGATACAAGCAAAA | 169 |
| *secF* | CCAGCAAATCAGGCAGAA | CTGCGTTGTTTGGCAATT | 176 |
| *ompR* | TTTATGTTGCCCGTTGAAGA | GTGCTAAAAGTTCATTTGGG | 178 |
| *nfuA* | TATGGCATATAGTGCACCAGA | CGCGGCACTTTAGAGTTT | 180 |
| *adeK* | CACAGAACAACCAGCTTCCA | TTGAGTCGAATCACGAGCAC | 212 |
| *mdfA* | TCGGTGTAAGTGCAACTTGG | GTTCAATTTGCCGCGTTAAT | 180 |
| *RS02655* | TTGAACAAAGCGTTGAGCTT | GTAGGAGTGATGCTTTTGCA | 190 |
| *RS02660* | GTGATGCCATGAAAGCAATG | TTGCACCAATGACACCAAGT | 237 |
| *RS00745* | ATGAATAAACCTTTCGACCAAG | TGATTTGACGCCATATGCT | 183 |
| *RS09720* | TCTGCCGACTTAAAACACTTTA | ATTGAATACGCAATTTTTACGC | 164 |
| *carO* | ATCCAAAACGACCAAGATGC | ACGAAAGTAGCGCCAACACT | 173 |
| *ftsK* | TATTCCGCATTTATTAACGCC | TCGGGTCAATCAAGTCTTCT | 185 |
| *recA* | CACGCCCTAGACCCTCAATA | CACCCATCTCACCTTCGATT | 193 |
